# Deep learning and generative AI for medical imaging and clinical decision support systems: a structured critical review

**DOI:** 10.3389/fdgth.2026.1890072

**Published:** 2026-08-18

**Authors:** A. Mohan Babu, A. Jeshurun Nehemiah, V. Jagadeep, R. Adaline Suji, Priyanka Nallusamy

**Affiliations:** School of Computer Science and Engineering, Vellore Institute of Technology, Vellore, India

**Keywords:** clinical decision support systems, convolutional neural networks, deep learning, diffusion models, generative AI, large language models, medical imaging, reporting guidelines

## Abstract

Deep learning (DL) and generative artificial intelligence (generative AI) are changing how medical data are analysed and used at the point of care. Our evidence base comprises 80 sources 37 screened studies and 43 landmark primary studies, architectural papers and clinical-AI reporting standards published between 2014 and 2026, across two related domains: medical image analysis and clinical decision support systems (CDSS). We trace the evolution from convolutional neural networks (CNNs) to U-Net and encoder–decoder networks, then to Vision Transformers (ViTs), generative adversarial networks (GANs), diffusion models, large language models (LLMs) and retrieval-augmented generation (RAG). Each model family is compared across nine dimensions: input modality, task, data requirements, validation level, interpretability, failure modes, clinical readiness, regulatory considerations and human-oversight need. Supervised DL reaches clinically useful performance across CT, MRI and pathology on well-scoped detection, segmentation and classification tasks, though results are task-, dataset- and site-dependent and prospective evidence is limited. To address data scarcity, generative models can produce synthetic images or cross-modality translations, but may amplify hidden dataset biases and generate anatomically incorrect images. LLM-based CDSS show promise for guideline-concordant reasoning and medication-safety checks, yet still face hallucination, calibration and regulatory uncertainty. For each technology we provide a deployment-readiness map, review the reporting standards needed for credible clinical evaluation (CONSORT-AI, SPIRIT-AI, TRIPOD + AI, CLAIM, DECIDE-AI, STARD-AI, PROBAST + AI, FUTURE-AI) and set out a research agenda centred on validation, governance and human-in-the-loop deployment. Unlike prior surveys, which treat imaging AI or clinical LLMs separately, we assess both, add an explicit evidence-quality appraisal and link each technology to the reporting standards. On current evidence, we argue that LLM-based CDSS are not supported for routine autonomous use and require clinician supervision. This is not a PRISMA-style systematic review but a structured critical narrative review built on a curated, transparently reported evidence base.

## 1 Introduction

### 1.1 Background and context

The digitalization of healthcare has resulted in a wealth of data coming from radiology studies, Electronic Health Records, (EHRs), genomics tests, and telemetry monitoring. Within this data rich context, AI and in particular deep learning (DL) has emerged as the most powerful paradigm for data analysis over the last few years, supplanting hand-crafted feature engineering with learned and end-to-end representations optimized for clinical functions (1, 2). CNNs were very successful in large-scale visual recognition, and were quickly applied to medical imaging, with reported accuracy levels rivalling those of expert radiologists and pathologists on specific limited tasks in a number of medical contexts (3, 4). The encoder–decoder networks, like U-Net and its variants, laid a solid foundation for pixel-wise segmentation and achieved accurate segmentation of anatomical structures and lesions in volumetric image collections (5, 6). However, several well-known challenges have hindered the clinical translation of DL models, such as the requirement of large, curated and labelled datasets, susceptibility to distribution shift between scanners and patient cohorts, and lack of transparency in model reasoning that limits interpretability (7, 8). These limits led to a second generation of approaches – generative AI – which include models that can not only interpret data but also generate and reason about it, thereby expanding the scope of clinical AI's operation (9–11).

### 1.2 Generative AI: an expanding frontier

Generative AI in healthcare encompasses generative adversarial networks (GANs), variational autoencoders (VAEs), denoising diffusion probabilistic models and large language models (LLMs). The applications of GANs have been used for augmentation and cross-modality translation/super-resolution problems where the problem is limited amount of labelled data and added heterogeneity in training sets (12–14). In a number of cases reported, diffusion models can generate higher fidelity and more diverse samples than GANs, and are becoming more popular for synthetic clinical data generation (15). Language reasoning is used to support clinicians in various ways, such as in guideline-based recommendations, expressing differential diagnoses, and highlighting potential medication-safety problems (16–19). Considering the implications of multimodal workflows, combining visual and textual models is a logical next step in medical AI, but with it come commensurate concerns of hallucinating content, bias, privacy, and the need for prospective validation for use in high-stakes environments (20–22).

### 1.3 Motivation and originality of this review

There are already good surveys of DL in medical imaging (1–4) and of LLMs in clinical medicine (21, 23). The contribution of this review, however, is not to re-catalogue those literatures, but to compare side by side imaging models and clinical-AI systems and evaluate them within a clear, explicit analytical framework (Section 2.5). Specifically, it is novel in four ways: (i) it applies a shared 9-dimensional comparison across all types of CNNs, U-Net variants, ViTs, GANs, diffusion models and the LLM/RAG-based CDSS models; (ii) it explicitly distinguishes between imaging AI and CDSS as well as when an imaging model is not a CDSS (Section 3.5); (iii) it provides a deployment-readiness map that separates capability from clinically proven readiness (Figure 3); and (iv) it incorporates the clinical-AI reporting-standards landscape (Section 6) into the evaluation, which most technology-focused surveys do not. A point-by-point comparison against the most closely related prior reviews, showing what each does and does not cover, is given in Section 5.5 (Table 1). The 2014–2026 window allows us to access more recent studies on the use of LLM in clinical specialties (21, 24, 25), diffusion-based imaging (15) and new governance ideas (20, 22) that pre-2023 reviews have been unable to address.

**Table 1:** Comparison of this review with the most closely related prior surveys.

Prior review (Type)	Imaging AI	Generative imaging	LLM / RAG-CDSS	Predictive AI-CDSS	Common analytical rubric	Evidence-quality / Readiness Appraisal	Reporting standards
Imaging DL Surveys (1–4, 26)	covered in depth	not covered	not covered	not covered	not covered	not covered	not covered
GAN in Medical Imaging (12, 13, 15)	partially covered	covered in depth	not covered	not covered	not covered	not covered	not covered
Generative AI Scoping Reviews (9, 10)	partially covered	partially covered	partially covered	not covered	not covered	not covered	not covered
Clinical LLM Reviews (21, 23, 44, 56)	not covered	not covered	covered in depth	not covered	not covered	partially covered	partially covered
Predictive AI-CDSS Meta-analysis (22)	not covered	not covered	not covered	covered in depth	not covered	covered in depth	partially covered
This Review	covered in depth	covered in depth	covered in depth	covered in depth	covered in depth (Nine-Dimension Framework, Section 2.5)	covered in depth (Supplementary Table S1)	covered in depth (Section 6, Table 5)

### 1.4 Principal contributions

This review makes four principal contributions. Evidence synthesis. We integrate evidence from medical image analysis and AI-based CDSS in a comparative framework, surfacing cross-domain insights that narrower surveys miss. Architectural evolution. We trace the shift from CNNs to U-Net variants, ViTs and LLMs, with comparative performance and explicit thresholds for clinical readiness (1, 3, 5, 26). Generative-model comparison. We contrast GAN-, diffusion- and LLM-based approaches on fidelity, safety, efficiency and clinical readiness (9, 12, 13, 15, 27). Research agenda. We identify directions in explainability, federated learning, human-in-the-loop evaluation and multimodality, framed by the reporting standards needed for credible clinical evaluation (20–22, 28).

## 2 Review methodology, scope and analytical framework

### 2.1 Type of review and rationale

This article is a structured critical narrative review. We do not claim the status of a systematic review as per PRISMA guidelines. 37 studies were selected to represent a core evidence base for the evolution and trade-offs of the field and supplemented with 43 landmark primary studies, foundation and methodological papers, reporting guidelines and supporting references for a total evidence base of 80 studies. We make this explicit because a systematic review of all the territory we address here (imaging DL, generative imaging, LLM-CDSS, predictive AI-CDSS, governance and regulation) would require thousands of records and a formal risk-of-bias appraisal that is too large to be addressed in a single integrative review. Search and selection are transparent as described below to permit readers to assess coverage and reproducibility, and to enable the work to be expanded to a systematic review if so desired.

Correction: This version of the manuscript originally contained a description of a “PRISMA-compliant systematic approach”, which was a translation of a section of the original paper. That statement has been rescinded. This article is not a PRISMA compliant or systematic review. The databases used, search strings, the last search date, eligibility criteria, the record screening and selection process and the number of records included in the analysis, and the quality appraisal process have been expanded in Sections 2.2–2.4 to allow others to assess the coverage of the review and re-run the review.

### 2.2 Information sources and search approach

Literature for candidates was retrieved from large bibliographic databases (PubMed/MEDLINE, IEEE Xplore, Web of Science and Scopus), and citation chasing of relevant reviews. “*Searches combined technology concepts with application concepts* such as “deep learning” and “medical imaging”, “diffusion model” and “medication safety”, and “U-Net” and “clinical decision support”. Records were pulled from various databases, deduplicated and then screened by title and abstract and full text against the following eligibility criteria and included in the search from 2015 to 2026.

#### Search strategy

PubMed/MEDLINE, IEEE Xplore, Web of Science and Scopus were searched for relevant records, and the reference lists of pertinent reviews were followed up (citation chasing). This final search was run on 30 June 2026. Records in English in 2014–2026 were searched. The basic Boolean search used in PubMed/MEDLINE was as follows: (“deep learning” OR “convolutional neural network” OR “U-Net” OR “vision transformer” OR “generative adversarial network” OR “diffusion model” OR “large language model” OR “retrieval-augmented generation”) AND (“medical imaging” OR radiology OR pathology OR segmentation OR “clinical decision support” OR CDSS OR “medication safety”) AND (clinical OR medical OR health). Field tags and syntax are adapted so as to be equivalent to the strings provided in the main text and included in Supplementary Material S2.

### 2.3 Eligibility criteria

Included: peer-reviewed journal articles or archival conference papers that discuss DL or generative AI applications to medical image analysis or CDSS, English language, adequate methodological description to allow for appraisal. The curated core ranges from 2017 to 2026, and a small number of foundational and landmark works from 2014 to 2016 (e.g., seminal architectures, early clinical-validation studies) have been included as supporting references, resulting in a total range of 2015–2026 matching the abstract**.** Excluded: Non-peer-reviewed papers other than clearly identified preprints, editorials with no primary or synthesised evidence, and papers that are not within the scope of imaging/CDSS. Preprints (when available) are marked explicitly in the reference list. Very recent (2025–2026) items were checked for indexing and publication status against the publisher record as the field is very dynamic.

### 2.4 Selection process and composition of the evidence base

**Figure 1:**
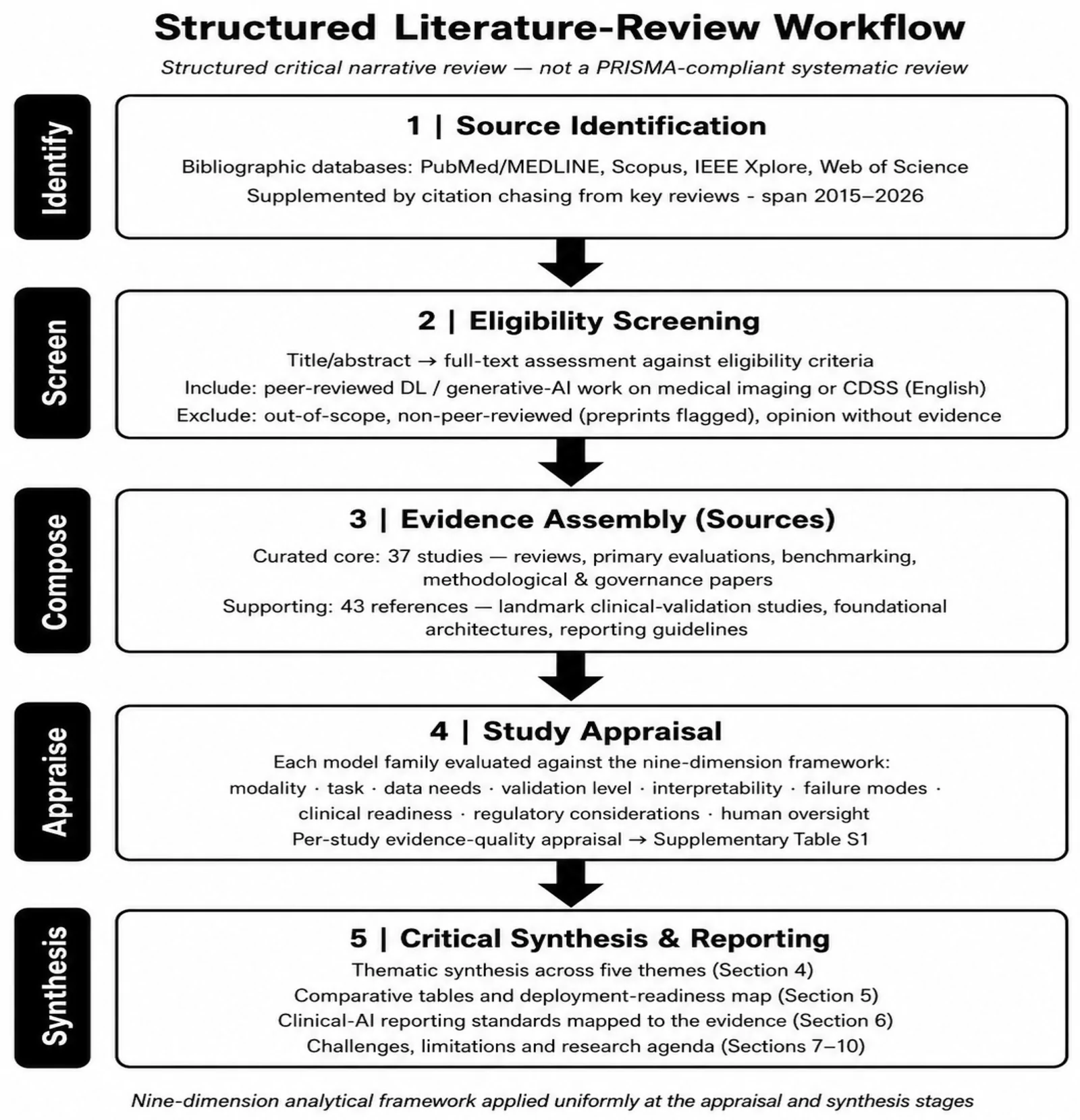
Structured literature-review workflow used in this study, from source identification and eligibility screening to thematic organisation, appraisal against the nine-dimension framework, and narrative synthesis.

Records retrieved from the four databases were pooled and de-duplicated, then screened first by title and abstract and subsequently at full text against the eligibility criteria set out in Section 2.3.The 80 studies included in the curated core are diverse from one another, including narrative, scoping and systematic reviews, primary evaluation and benchmarking studies, methodological or governance papers. For context and to facilitate critical appraisal, the review also includes 43 supporting citations, primarily from key architectural works and clinical validation studies that are essential to the understanding of the core, but not included in the screened core; these are analytical, not screening, citations. The core is broken down per study, with information on the study type, validation design, dataset scope, external-validation status, and an indicative clinical-readiness tag provided in Supplementary Table S1, which is required for a critical review. Figure 1 explains the Structured literature-review workflow used in this study, from source identification and eligibility screening to thematic organisation, appraisal against the nine-dimension framework, and narrative synthesis.

#### Selection process

The records from the databases were deduplicated and, based on the eligibility criteria, screened by title and abstract. Full-text evaluation was performed on potentially relevant articles. From this, a subset of 37 studies was selected. An additional 43 key primary studies, seminal architectural papers and reporting guidelines were added to the evidence base as analytical (non-screened) supporting references, bringing the total to 80 sources. The overall literature identification and literature selection process is summarized in Figure 1.

### 2.5 Analytical framework

**Figure 2:**
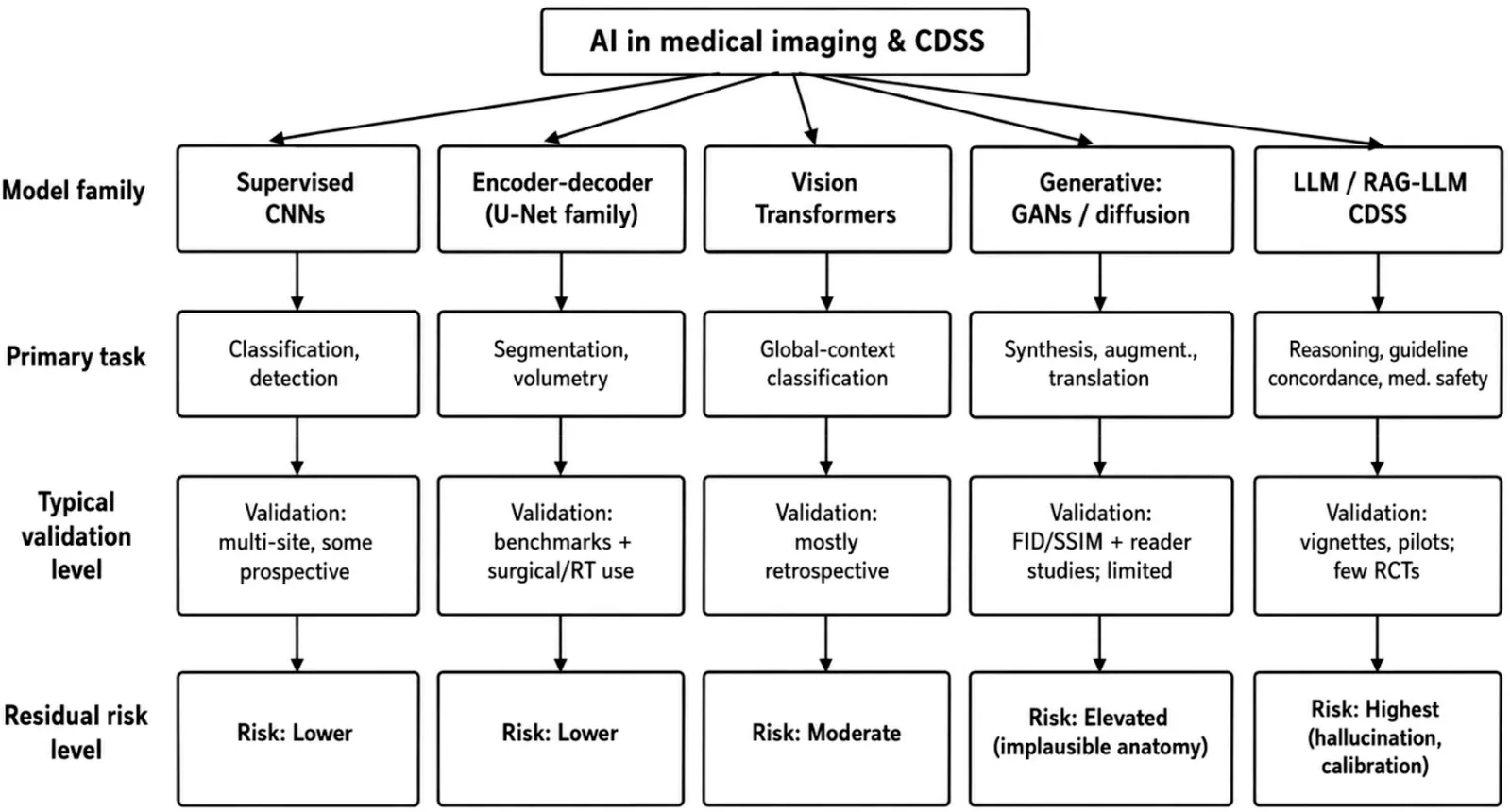
Taxonomy of the model families reviewed, mapped onto their primary task, typical validation level and residual risk.

In order to be able to go from description to analysis, each model family is analysed on the same 9 dimensions, and these dimensions structure Tables 2–4 and the taxonomy in Figure 2:

**Table 2:** Thematic summary of the 37 included studies (2017–2026). Counts are approximate groupings; several works span themes.

Theme	Studies (n)	Representative references	Dominant study type	Maturity/clinical readiness
DL in medical imaging	**8**	Lee (1); shen (3); chan (4); sistaninejhad (26); wiestler (49); Ul Haq (46); tajbakhsh (5); miotto (31)	Reviews + benchmarking	High for scoped tasks; some multi-site and prospective use
Generative imaging (GAN/diffusion)	**6**	Yi (12); skandarani (13); zhou (14); kazuhiro (15); moulaei (9); bhuyan (10)	Reviews + empirical comparisons	moderate; reader studies, limited prospective validation
Generative AI in health systems	**5**	moulaei (9); bhuyan (10, 20); zhang (11); Ong (27)	Scoping reviews + governance	Emerging; governance-focused
LLMs in CDSS	**9**	vrdoljak (23); shool (44); tofeeq (24); oniani (16); hager (17); Li (25); zhang (59); Ong (18); Tara (19)	Benchmarking + systematic reviews	Early pilots; few RCTs
Predictive/disease-specific AI-CDSS	**3**	elhaddad (45); meta-analysis (22); bajramagic (28)	Meta-analysis + prospective	deployed in select centres; prospective evidence growing

**Table 3:** Technical comparison of DL and generative-AI architectures.

Model type	Representative architectures	Primary application	Indicative performance (task-dependent)	Key strengths	Key limitations/source
CNN (supervised)	ResNet, VGG, DenseNet, EfficientNet	Classification; lesion detection	AUROC ≈ 0.85–0.97 on specific radiology tasks (1, 3, 4)	Proven on scoped tasks; efficient; understood failure modes	Distribution-shift sensitivity; annotation-dependent (46)
Encoder–decoder (U-Net)	U-Net, U-Net++, Attention U-Net, Swin-UNet	Semantic segmentation (organ, tumour)	Dice ≈ 0.80–0.95 for organ segmentation (5, 6, 26)	Precise localisation; effective under limited labels	Volumetric complexity; boundary edge cases
Vision Transformer	ViT, Swin Transformer, TransUNet	Global-context classification; hybrid segmentation	Competitive with CNNs on large datasets (8, 26)	Long-range dependencies; interpretable attention	Data-hungry; high compute; limited small-set benefit
GAN (generative)	CycleGAN, DCGAN, StyleGAN-Med, SR-GAN	Synthesis; augmentation; super-resolution	FID ≈ 15–80 (domain-dependent); SSIM ≈ 0.87–0.96 (13, 14)	Addresses scarcity; cross-modal translation	Instability; hallucination risk; metric disagreement (13, 15)
Diffusion model	DDPM, latent diffusion (medical adaptations)	High-fidelity synthesis; denoising; reconstruction	Reported superior mode coverage vs. GANs; validation emerging (9, 15)	Stable training; diverse outputs	High inference cost; limited clinical validation
LLM (general)	GPT-4, Claude, Gemini, Llama variants	Medical Q&A; documentation; differential diagnosis	Reported USMLE-style pass rates ≈ 80%–90% (23, 24)	Broad knowledge; few-shot; multimodal	Hallucination; calibration gaps; opaque reasoning
LLM (domain-adapted)	Med-PaLM, BioGPT, Clinical-LLaMA	Guideline-concordant reasoning; medication safety	Reported gains over general LLMs on clinical benchmarks (16, 18)	Better guideline adherence; specialty calibration	Training cost; guideline-update lag; few specialties
RAG-augmented LLM	LLM + retrieval (guidelines, EHR)	Real-time guideline integration; recommendations	Improved guideline concordance vs. standalone LLM (16)	Currency; source attribution; audit-traceable	Retrieval-quality dependent; latency; complex deployment

**Table 4:** Trustworthiness and efficiency of AI systems in healthcare.

System category	Explainability	Reliability/calibration	Compute cost	Clinical readiness	Human-oversight need
CNN-based imaging (1–4, 26)	Moderate (Grad-CAM, saliency)	High on validated benchmarks	Low–medium (inference)	High (deployed in radiology)	Low–moderate
U-Net segmentation (5, 6)	Moderate (attention maps)	High in-distribution	Medium	High (surgical planning, RT)	Moderate
Vision Transformers (8, 26)	Good (attention viz.)	Moderate (data-hungry)	High (training)	Emerging	Moderate
GAN synthesis (12–15)	Low (black-box generator)	Variable (hallucination risk)	High (training)	Limited (regulatory unclear)	High
Diffusion models (9, 15)	Very low	Moderate–high (mode coverage)	Very high (inference)	Research stage	High
General LLMs (clinical) (23, 24, 44)	Very low	Variable (calibration gaps)	High (API/GPU)	Limited (regulatory pending)	Very high
Domain-adapted LLMs (16, 17)	Low–moderate	Better than general LLMs	High	Emerging (pilots)	High
RAG-augmented LLM (16)	Moderate (source attribution)	Good (guideline-grounded)	High (retrieval + inference)	Promising	High
LLM-CDSS (specialty) (18, 19, 25)	Low (opaque reasoning)	Variable (task-dependent)	High	Early clinical pilots	Very high
Predictive AI-CDSS (22, 28, 45)	Moderate (SHAP, LIME)	Good (prospective validation)	Low–medium	Deployed in select centres	Moderate

The following were considered: (1) input modality, (2) output/task, (3) data requirements, (4) typical validation level (retrospective, benchmark, reader study, prospective/RCT), (5) interpretability, (6) characteristic failure modes, (7) clinical readiness, (8) regulatory considerations, and (9) human-oversight requirement. A fixed rubric allows for the comparison to be reproduced and reveals a trend (explained in Section 9.2) that as capability increases, reliability is sacrificed, and vice versa, throughout the model continuum.

## 3 Background and fundamentals

### 3.1 Deep-learning architectures

#### 3.1.1 Convolutional neural networks (CNNs)

CNNs capture spatial structure hierarchically, using stacked convolution, pooling and non-linear activation. Trainability and efficiency have been improved by residual connections (ResNet), dense connectivity (DenseNet) and depth-wise separable convolutions (MobileNet). Improvements in deep representation learning (29) spurred the modern era and large initial surveys reported the rate of CNN adoption in radiology, histopathology and ophthalmology (30). Medical imaging has benefited greatly from CNNs, with their role in classification being evident in histopathology and radiology, as well as in retinal disease; they are also the architectural basis for most systems reviewed here (1, 3, 31).

#### 3.1.2 Encoder–decoder architectures and U-Net

Biomedical image segmentation was the motivation for U-Net (32) which employed long skip connections between the encoder and decoder to combine local and global features. It is modular in nature, and has inspired numerous variants (Attention U-Net, U-Net++, Swin-UNet) that continue to be top choices for volumetric segmentation (5, 6). In particular, self-configuring pipelines like nnU-Net (33) have demonstrated that a disciplined approach of preprocessing and training heuristics can achieve similar or superior architectural novelty when tested on dozens of segmentation benchmarks, which is a warning against assuming that newer architectures necessarily outperform their predecessors in clinical performance. This has been driven by good performance with low labelling cost and has facilitated its application in clinical applications like surgical planning and radiotherapy contouring.

#### 3.1.3 Vision transformers (viTs)

In contrast, CNN inductive biases are unable to provide global context, which is achieved by the self-attention mechanism (34) of NLP used in ViTs (35). CNN–Transformer hybrids (such as TransUNet (36) and hierarchical Transformers (Swin Transformer) have received competitive performance in medical-imaging applications and can enhance the interpretability by using attention maps, but are generally more data- and computation-heavy than CNNs and do not always outperform well-tuned CNNs in the smaller-scale regimes typical of medical imaging (8, 26).

### 3.2 Generative-AI models

#### 3.2.1 Generative adversarial networks (GANs)

Adversarial learning which is a minimax game between generator and discriminator is used by GANs (37) to create samples that are hard to tell apart from real data. In health care they have been used in image synthesis, domain adaptation and super-resolution, and, to create anonymized synthetic images. Training may be unstable (mode collapse) and there is a risk of hallucinated pathology, or image features not grounded in anatomy (12–15).

#### 3.2.2 Diffusion models

High fidelity, diverse images are created using denoising diffusion probabilistic models (38) which create images with a gradual noising process followed by a reverse process. They can model training distributions more realistically than GANs, and have been adopted widely for medical-image generation: latent-diffusion variants (39) do so by working in a latent space that is compressed, and a survey (40) lists and describes the myriad medical uses that are growing rapidly. Despite this, inference still remains a costly process, as many denoising steps are needed per sample, thereby restricting the use of inference in resource-limited environments (9, 15).

#### 3.2.3 Large language models (LLMs)

LLMs are transformers that are trained on extremely massive text corpora that demonstrate emergent properties of multi-step reasoning, differential diagnosis, and procedure generation. Medical literature (and EHRs) models have achieved expert-level performance on medical-licensing style benchmarks (41, 42) and there has been a proposal for a paradigm shift in the field with the introduction of generalist medical foundation models trained across imaging, text and structured data (43). But benchmark performance has been repeatedly found to be a poor indicator of bedside utility: hallucination, calibration, and safe use in high stakes environments are still active research issues (16, 17, 24, 44).

### 3.3 Medical-imaging modalities

This review covers computed tomography (CT) in lung cancer and abdominal tumour detection, and in conditions of trauma; magnetic resonance imaging (MRI) in neurological, musculoskeletal and cardiac imaging; point-of-care ultrasound, increasingly analysed on mobile devices using DL, in tumour detection; and whole-slide images (WSI), in digital pathology, for histopathological classification.

### 3.4 Clinical decision support systems (CDSS)

CDSSs are interactive computer-based systems that are used to enhance the performance of a clinician by providing knowledge or information specific to a particular clinician (22). The classical model is generalised by introducing representations learned from clinical corpora instead of rule-based knowledge, with the help of AI-based CDSS. Today's LLM-generated CDSS can process the imaging, free text, and structured EHR data, and provide ranked differential diagnoses, identify medication-safety issues, and identify supporting evidence (22, 28, 45).

### 3.5 Distinguishing imaging AI from CDSS

Although related, the fields of Imaging AI and CDSS are not the same, which is why it is important not to confuse them and the level of evidence that's needed. As we proceed we will be working with the following distinctions. When an imaging model produces a measurement or classification (such as a nodule probability or segmentation mask) that a clinician later interprets, it is a stand-alone diagnostic model. When the output of the same model is coupled to a workflow that provides a patient specific recommendation or alert (e.g., triage prioritisation, treatment suggestion), the model becomes a CDSS component. LLM-based CDSS are not necessarily discriminative models as per predictive AI-CDSS, which produce a calibrated risk score suitable for prospective validation against outcomes (TRIPOD + AI-style development and validation reporting), but rather generate free text and thus require extra measures to guard against hallucination and mis-calibration, as well as reader-facing verification (DECIDE-AI-style early clinical evaluation). The evidence bar is raised from a stand-alone measurement model to a predictive alerting CDSS to a generative recommendation CDSS.

## 4 Structured synthesis of the literature

The evidence has been divided into five themes. Studies are not summarised in isolation, but methodologies are compared; tensions and findings are noted when they conflict; and ideas and tensions are tracked across the 21st century window 2017–2026. Section 2.5 provides the framework for each theme to be read.

### 4.1 Deep learning in medical imaging

#### 4.1.1 Foundational architectures and early applications

The theoretical and empirical works of Lee et al. (1) and Shen et al. (3) set the stage for CNNs in medical imaging, demonstrating that CNNs could compete with expert-level accuracy on a small set of tasks, including chest-radiography classification and brain-lesion segmentation, but mostly in a retrospective and single benchmark manner. Chan et al. (4) did this for radiology workflows, however, highlighting the considerable amount of preprocessing, calibration and validation that must be done for integration. Zhou et al. (2) introduced an early notion that imaging properties (resolution, dimensionality, noise, etc., and how they vary during acquisition) influence the definition of models, definition of loss functions, and evaluation—a concept that has persisted. Taken together, they provide a conceptual framework for the literature to follow, but also highlight the early use of retrospective evaluations in the field, a practice which is repeated throughout this review.

#### 4.1.2 Segmentation: U-Net and its descendants

The use of segmentation has been consistently studied due to its applications in surgery planning, radiotherapy targeting and lesion volumetry. Tajbakhsh et al. (5) discussed DL segmentation in the presence of imperfect labels, which suggest that transfer and semi-supervised learning can partially compensate for the lack of annotations. Sistaninejhad et al. (26) compared U-Net, U-Net++ and ResU-Net with different datasets and targets and a review in (6) matched different segmentation loss functions with clinical goals (Dice, focal, Hausdorff-distance). One consistent theme is the use of attention and Transformers in encoder–decoder architectures: Swin-UNet and TransUNet use the ViT-based decoders to model long-range spatial correlations which is crucial for lesions with indistinct margins, while preserving skip-connection details (26, 46). Promptable foundation segmentation models (47) and its medical variant MedSAM (48) have recently started the trend towards general-purpose, promptable models, but their performance on specialised modalities remains inconsistent in the zero-shot setting and often needs tuning to perform better in a medical context. The disagreements come in on how much of these advances extend beyond the curated benchmarks, which is not yet well documented.

#### 4.1.3 Classification, detection and multimodal integration

In addition to segmentation, DL has been tested for classification and detection. Wiestler (49) gave a tutorial focused on training, validating and interpreting CNN classifiers for use in neuro-oncology, highlighting the disconnect between benchmark performance and clinical application. Ul Haq (46) reviewed architectures on the topic of distribution shift (performance loss from using the model at a different site than it was trained on) and proposed domain adaptation and federated learning. Miotto et al. (31) classified imaging DL as part of multimodal healthcare AI, noting that multimodal models that incorporated imaging and structured EHR information (laboratory information, demographics) generally achieved better performance on downstream tasks. The early multimodal implementations that followed, reinforced by the foundation-model implementations (50) form the basis of the hybrid CDSS designs outlined in Section 4.4. The promise and the empirical limits of imaging DL have been illustrated by a few landmark primary studies; for example, dermatologist-level skin-cancer classification achieved expert comparable accuracy in large curated datasets (51) and the automated detection of diabetic retinopathy (DR) was shown to be expert-comparable accuracy on large curated datasets (52); the latter was subsequently expanded to screening populations for breast cancer (53) and lung cancer (54). However, most of these assessments have been reader-study or retrospective in nature, and have been shown to fail to attain the same levels of accuracy when applied to unselected external multi-site populations, as noted throughout this review, and which is frequently seen in external validation of these models. The development of imaging foundation models like RETFound (55) that were trained self-supervisedly on millions of retinal images suggest a future direction towards more label efficient and transferable representations, which is still to be demonstrated in the context of clinical usefulness.

### 4.2 Generative models in medical imaging

#### 4.2.1 GANs: promise, performance and pitfalls

Yi et al. (12) made a systematic review of the medical-imaging GANs and found that augmentation and cross-modality translation (e.g., CT-to-MRI) were most effective in the label-scarcity problem. Skandarani et al. (13) then empirically compared GAN variants (DCGAN, CycleGAN, StyleGAN adaptations), where no single architecture was found to outperform the rest and where quantitative metrics (FID, SSIM) sometimes disagreed with radiologist scoring, especially a word of caution against just relying on purely quantitative measures to assess synthesis quality. Kazuhiro et al. (15) provided a radiologist-oriented introduction to CT dose reduction (denoising GANs), MRI artefact reduction and regulatory considerations of the use of synthetic images for diagnosis, highlighting that GANs can yield “hallucinated” lesions that are radiologically plausible but not anatomically correct, which could pose a patient-safety risk. Ahmad et al. (14) previously introduced a GAN-based super-resolution system to help enhance the diagnostic confidence of low dose CT, which demonstrated the potential and the non-negligible uncertainty in generative imaging.

#### 4.2.2 Diffusion models and beyond

Diffusion models are a growing generative approach in healthcare, with several works using them as such (9, 15). Bhuyan et al. (10) and Moulaei et al. (9) observed the increasing adoption of diffusion-based synthesis in radiology and pathology, where high-fidelity, and diverse synthetic data are required for pre-training. Although diffusion models possess theoretical benefits (high model coverage, training stability, tractable likelihood), some researchers have thus far cast doubt on the possibility that diffusion models might eventually replace GANs for medical-image synthesis, and this is tempered by an order of magnitude higher inference cost that affects heavily compute-constrained applications.

### 4.3 Generative AI in healthcare systems

#### 4.3.1 Landscape and applications

Moulaei et al. (9) offer the most comprehensive scoping review, covering imaging, clinical-text generation, patient communication, administration and education; with a clear domination in imaging or clinical text generation and a smaller volume of studies in multimodal pipelines. Bhuyan et al. (10) introduced an institutional-governance analysis, which states that regulatory frameworks for high-stakes generative AI must be more complex than those for deterministic devices. Zhang and Kamel Boulos (11) explored the application of generative AI in primary care and found it to have potential applications for documentation (AI-generated SOAP notes), patient engagement (AI-generated responses), and support with differential diagnosis (AI-generated suggestions). They noted that it has the potential to reinforce systematic error if used passively and without critical awareness. Bhuyan et al. (20) set this in an ethical and legal accountability framework which requires disclosure, informed consent and audit trails as minimum requirements.

#### 4.3.2 Medication safety and harm reduction

AI assisted detection of drug interactions, contraindications and dosing errors in polypharmacy were cited in a scoping review of generative AI and LLMs for medication safety (27). LMs trained on medical corpora performed strongly on common, well-represented drug-interaction queries, including in zero-shot settings. They failed, however, on rare interactions and on guidelines published after their training cut-off — precisely the situations in which human oversight is most needed. This drives the “human in the loop” design of Section 6 and research directions of Section 8.

### 4.4 Large language models in clinical decision support

#### 4.4.1 Performance across clinical benchmarks

Since 2023, there has been a growing number of LLM evaluations for CDSS, but a systematic review of health-care LLM applications revealed that a vast majority of the evaluations focused on accuracy or exam-type tasks, versus on actual patient-care processes or safety and equity endpoints, and that the practice of evaluating LLMs was inconsistent (56). Vrdoljak et al. (23) reported that LLM performance in education and CDSS/administration was good on the knowledge-retrieval tasks (such as answering questions in medical Q&A, licensing exams) and was mixed when it came to reasoning that requires multiple patient-specific data points. Shool et al. (44) analysed the evaluation practices in 87 LLM clinical studies and revealed significant variation in the benchmarks, metrics and reporting methods—variation which will make it difficult to compare clinical studies in terms of their benchmarks and metrics, as well as report the results, and which will justify reporting standards discussed in Section 7. Tofeeq et al. (24) compared several LLMs (GPT-4, Claude, Gemini, Llama variants) on a medical Q&A task and found that while the frontier models were able to pass the licensing exam, they were significantly miscalibrated in medical subspecialties, and that a model which assigns high confidence to answers that are wrong is considerably more dangerous than one that signals its own uncertainty. Domain-adapted medical LLMs, such as Med-PaLM 2 (57), have further improved licensing-exam performance which, however, remains a poor indicator for open-ended, safety-critical reasoning in the clinic.

**Figure 3:**
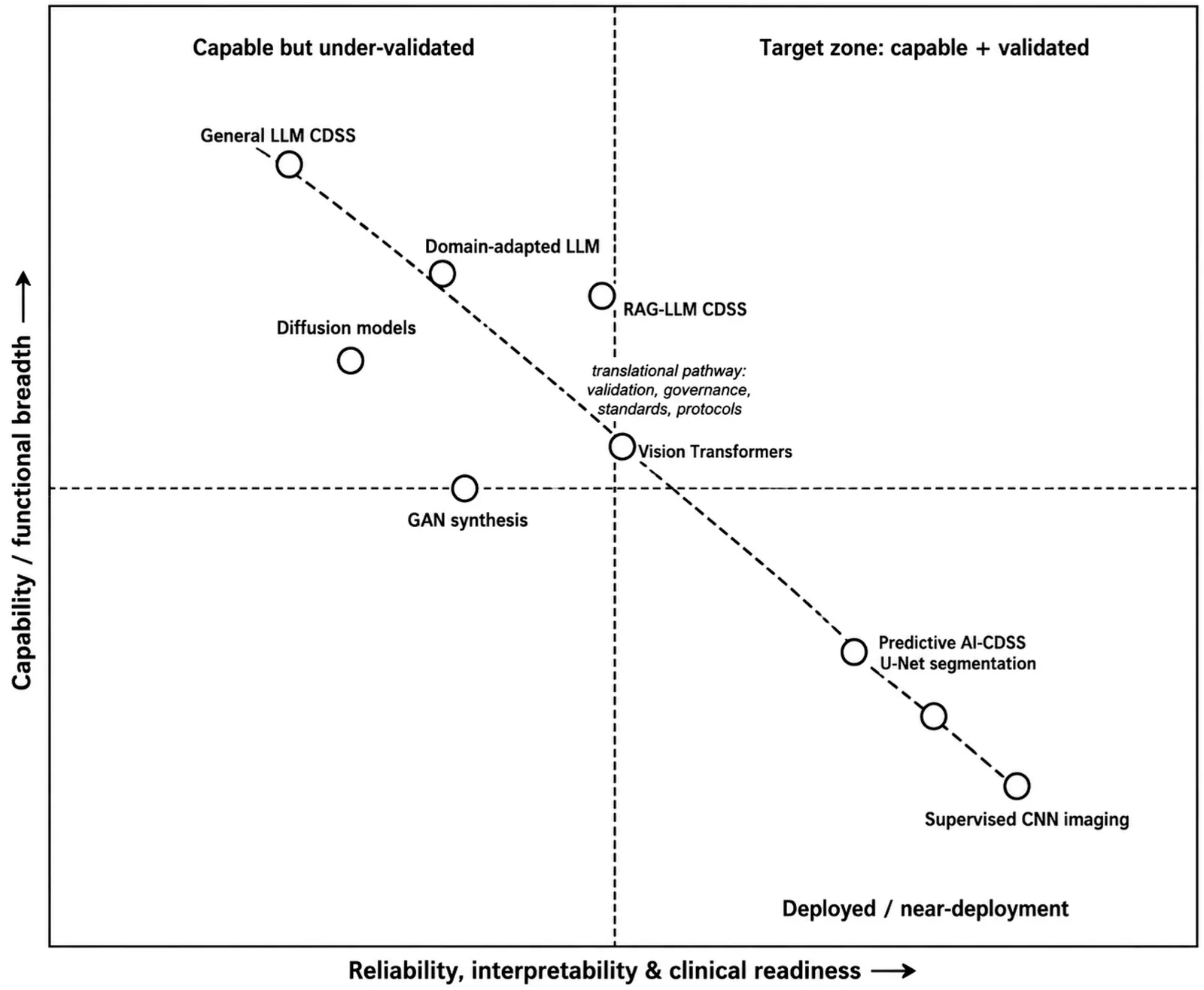
Deployment-readiness framework for the surveyed model families.

Figure 3 shows the deployment-readiness framework for the surveyed model families. The horizontal axis captures reliability, interpretability and validated clinical readiness; the vertical axis captures functional breadth. Supervised imaging models and predictive AI-CDSS occupy the deployed/near-deployment region; generative and LLM-based systems are more capable but under-validated. The dashed arrow marks the translational pathway (validation, governance, human–AI protocols) rather than raw capability gains.

#### 4.4.2 Guideline integration and reasoning enhancement

Oniani et al. (16) identified poor consistency of general-purpose LLMs with existing guidelines as an important limitation, and they showed that adding a retrieval-augmented generation (RAG) (58) system to augment structured guidelines at inference time leads to significantly higher adherence to guidelines in simulated vignettes compared to using a standard prompt. Challenging this with an empirical study, Hager et al. (17) tested state-of-the-art LLMs with 2,400 real patient cases from the intensive-care unit records of four abdominal diseases; models outperformed physicians in accuracy, but failed to adhere to medical guidelines, failed to interpret lab results, and were sensitive to the order and amount of information, leading the authors to conclude that current LLMs are not ready for autonomous clinical decision making, requiring careful human oversight. But a big synthesis of the clinical-LLM literature (21) came to a unanimous conclusion: LLMs are better at extracting structured knowledge, less good at reasoning about unstructured knowledge, and, screening 4,609 papers from 2022 to 2025, few used real-world patient data and only 19 were prospective, randomised, controlled trials, which is why the evidence base is so thin.

#### 4.4.3 Specialty-specific CDSS deployments

There is variation across specialty studies in the nature of the tasks. To assess the capabilities of the three frontier LLMs in real internal-medicine cases, Zhang et al. (59) compared their performance on differential diagnosis tasks and observed that GPT-4 performed better in these cases but was more likely to suggest inappropriate treatment plans in complex pharmacological cases; Li et al. (25) investigated whether LLM-powered support improves or competes with human ability, and they also concluded that current evidence favors supervised augmentation over autonomous use. The results showed that, in a prospective, cross-over study with 91 medication safety scenarios across 16 specialties, a pharmacist-plus-LLM combination achieved a 32% increase in DRP detection as compared to pharmacists alone, but the LLM alone performed less effectively, suggesting that the LLM should augment rather than replace pharmacists. In a prospective, cross-over study involving 91 prescribing-error scenarios across 16 specialties, Ong et al. (18) reported that the pharmacist-plus-LLM configuration detected approximately 32% more drug-related problems (DRPs) than pharmacists alone, whereas the LLM alone performed less well than pharmacists — evidence that favours augmentation of, rather than replacement of, the pharmacist.by LLM. Tara et al. (19) assessed the performance of LLMs in gastroenterology and hepatology, revealing that while LLMs provided recommendations consistent with guidelines during a structured endoscopy and histology input, the level of these recommendations was limited to that of trainees and below subspecialty hepatologists. All these studies support a limited scope deployment, meaning that there is a need for clinicians to be involved, rather than autonomous deployment.

### 4.5 AI-driven CDSS: predictive and disease-specific systems

In addition to LLMs, there have been many studies on predictive DL-CDSS. Elhaddad and Hamam (45) classified CDSS based on their way of representing knowledge (rule-based, probabilistic, DL-based, hybrid) and found that, while discrimination (AUROC, sensitivity, specificity) was often better with DL-based CDSS, they were less accepted because they did not explain their reasoning. A systematic review and meta-analysis of predictive AI-CDSS in 50 studies and 17 specialties (22) showed moderate pooled discrimination overall, with relatively high specificity, but a relatively low and less consistent sensitivity, and—most importantly—showed that most evaluations were performed on historical, retrospective data rather than tested in real clinical workflows, highlighting the mismatch between performances and real application. Among the few prospective clinical examples of the utility of AI-CDSS reported by Bajramagic et al. (28) better HbA1c attainment and reduced time to intensification were observed in LLM based systems.

## 5 Comparative analysis

This section presents three summary tables and then draws cross-cutting insights on performance trends, deployment readiness and the capability–reliability trade-off. The evidence-and-appraisal matrix for each study is included as a Supplementary Table S1, while Table 2 is a thematic summary of the evidence-and-appraisal matrix.

### 5.1 Thematic summary of the evidence base

There are three trends that stand out. The first is a movement from “imaging” CNN studies (2017–2021) to “GAN/multimodal” studies (2019–2023) and finally to LLM-based CDSS (2024–2026), marking the trajectory of generative-AI innovation. Secondly, the increasing prevalence of CDSS studies following the widespread dissemination of frontier LLMs. Third, prospective clinical validation is lacking: data from most studies are retrospective, and most include benchmarks or reader panels but not randomised or prospective outcome evaluation—the translational gap revisited in Section 9.4. These themes are summarised in Table 2.

### 5.2 Technical comparison of architectures

The performance numbers that follow are indicative ranges reported in the literature surveyed and are highly task-, dataset- and modality-dependent and should not be interpreted as benchmarks for any particular clinical task. The ranges are traced back to the studies referenced in the last column and to Supplementary Table S1, one study at a time. If a range cannot be connected to a specific study that was surveyed, the range has been deleted or identified as an illustrative range.

The Table 3 reveals a design compromise between ability and reliability. At the other end of the spectrum, supervised CNNs, and U-Net, are comparatively well understood, easy to interpret, and have clinical pathways: ViTs have higher data and compute costs compared with smaller, single-site deployments, but have greater global context. Generative models (GANs, diffusion, LLMs) present novel capabilities (synthesis, reasoning, generalisation) but pose trustworthiness and regulatory and deployment challenges. A middle ground approach is the RAG-augmented LLM, where hallucination is significantly curtailed, but not eliminated, by grounding the generation in documents retrieved by RAG, while still offering the flexibility of reasoning (16).

### 5.3 Trustworthiness and efficiency

Table 4 depicts the trustworthiness and efficiency of AI systems in healthcare. Ratings are qualitative assessments synthesised from the surveyed evidence; clinical readiness reflects the degree of prospective validation and regulatory engagement rather than a formal certification status. A consistent pattern emerges when comparing the clinical readiness, explainability, and oversight requirements of the different systems; the more mature their algorithms are, the greater their clinical readiness and their explainability, but the lower their oversight requirements. The systems with the highest functionality generally exhibit the lowest explainability and calibration and require the most human supervision, namely LLM-based systems. It is not a coincidence, but is part of the nature of autoregressive models that they generate text, not through explicit clinical reasoning, but through probabilities at the level of tokens. For CDSS architects, the takeaway is that there needs to be a mandatory “human check” and audit trails, as well as some tooling for calibration, until explainability methods are improved.

### 5.4 Why particular architectures outperform others, and what still blocks deployment

The comparisons in Tables 3, 4 are not just a simple ranking – the differences in performance are understood within the context of the match between the inductive bias of an architecture and the structure of the clinical task. CNNs are still the most dominant models for scoped classification and detection problems, as they are naturally local and translation-equivariant, a property that fits well with the prevalent nature of focal pathology in images, and because they are also data-inefficient, a crucial advantage when medical datasets are orders of magnitude smaller than the billions of images on which they are typically trained (1, 3, 46). This is because a purely contractive encoder would lose all of the fine spatial detail required for boundary-accurate delineation, which is what U-Net retains by the use of skip connections, and for this reason, newer models often do not outperform a well-tuned baseline whose architecture may not be as novel as that of the model. This is because newer models tend to not outperform a well-tuned baseline, whose architecture may not be as novel as the model, but has its performance heuristics well controlled and disciplined; in the realm of segmentation, this discipline and heuristics are equally important as architectural novelty. The advantage of ViTs lies in the ability to capture a diffuse or ill-defined pattern or large-scale context (contextual awareness) but the weak inductive bias has to be paid for with data or large-scale pretraining, which is why their benefit is negligible for the small, single site regimes found in clinical practice (8, 26, 35). Diffusion models outperform GANs in mode coverage due to the lack of the mode collapse problem in training a generative minimax game and the need for only inference time. Last, RAG augmented LLMs do not perform better than standalone LLMs on the guideline-concordance task, since using a retrieved, up-to-date source as a substitute for parametric recall provides verifiable evidence for the generation of the task (16, 58). Any of these benefits must be overcome in the clinical world, given through three unresolved questions. First, it is task-, dataset- and site-specific: the ranking above relies on selected benchmarks and often does not hold up to scrutiny when confronted with external unselected populations, as the inability of many hundreds of published imaging models for COVID-19 to produce a single clinically useful one shows (60).

Second, the evaluation metrics themselves are disputed: FID and SSIM are in disagreement with radiologist judgement for synthetic images (13) and discrimination metrics do not relate to calibration (or how well an output can be acted upon safely) (17, 24). Third, there is no evidence threshold that defines when an architecture is “ready”; readiness depends on the task, workflow and jurisdiction, and even tools that have been cleared must be locally validated (Section 9.1). Instead, one should consider selecting an architecture to provide a match with inductive bias, data budget and oversight capacity to the clinical task, rather than the highest scoring model.

### 5.5 How this review differs from existing surveys

Several recent reviews cover parts of this territory, and it is reasonable to ask what a further review adds. Table 1 sets out the comparison explicitly. In brief: the imaging-focused surveys (1–4, 26) predate the generative era and do not address CDSS; the generative-AI scoping reviews (9, 10) map applications across healthcare but do not compare architectures against a fixed analytical rubric or appraise clinical readiness; the clinical-LLM reviews (21, 23, 44, 56) appraise LLM evaluation practice rigorously but exclude imaging entirely; and the predictive AI-CDSS meta-analysis (22) is confined to discriminative models. To our knowledge, no existing review (i) applies one common nine-dimension rubric across the full span from CNNs to RAG-based CDSS, (ii) treats imaging AI and CDSS as a single evidence landscape while stating explicitly where an imaging model is and is not a CDSS (Section 3.5), (iii) attaches an evidence-quality and clinical-readiness appraisal to every included study (Supplementary Table S1), and (iv) maps the technology onto the clinical-AI reporting-standards landscape (Section 6) to define a staged evidence pathway. The contribution is therefore integrative and evaluative rather than enumerative.

## 6 Reporting standards and evaluation frameworks for clinical AI

Reporting and evaluation frameworks, which form the basis of whether a medical AI claim is believable, are compulsory for a survey of deployable medical AI to be complete. Such frameworks aim to decrease the heterogeneity of LLM evaluation referred to by Shool et al. (44). Early meta-research echoed the need for these standards, with a systematic review of deep-learning studies claiming clinically equal performance showing numerous with poor study design, limited external validation, and exaggerated results (61). We thus summarise the standards that are most relevant to the systems reviewed here and where they apply as shown in Table 5.

**Table 5:** Reporting and evaluation frameworks relevant to deployable medical AI and CDSS, mapped to the parts of this review to which each applies.

Framework	Scope/what it governs	Relevance to this review
CONSORT-AI (74)	Reporting of randomised clinical trials of AI interventions	The bar for prospective LLM-CDSS and predictive AI-CDSS trials (Sections 4.4–4.5)
SPIRIT-AI (77)	Clinical-trial protocols for AI interventions	Pre-registration companion to CONSORT-AI for planned CDSS trials
TRIPOD+AI (78)	Reporting of prediction-model studies using ML	Predictive AI-CDSS (risk scores, deterioration/sepsis models) (22, 28, 45)
CLAIM (79)	Reporting of AI in medical imaging	Imaging DL, GAN/diffusion synthesis and segmentation studies (1–6, 12–15, 26, 46, 49)
DECIDE-AI (73)	Early-stage (live) clinical evaluation of AI decision support	First-in-clinic evaluation of LLM- and predictive-CDSS pilots
STARD-AI (80)	Diagnostic-accuracy studies of AI (in development)	Diagnostic imaging classifiers and diagnostic LLM tasks
PROBAST+AI (76)	Risk-of-bias and applicability appraisal of AI prediction models	Critical appraisal underpinning Supplementary Table S1
FUTURE-AI (71)	Trustworthiness principles across the AI lifecycle (fairness, universality, traceability, usability, robustness, explainability)	Cross-cutting governance for imaging AI and CDSS deployment

Two implications follow. One factor contributing to the lack of clinical utility from apparently good benchmark results is that the majority of the performance evidence that was reviewed in Sections 4–5 predates or does not follow these standards. Second, the standards themselves suggest a staged evidence pathway: starting with CLAIM/TRIPOD + AI compliant development reporting, moving forward to DECIDE-AI early clinical evaluation, to CONSORT-AI/SPIRIT-AI trials, and then onwards. FUTURE-AI provides human-in-the-loop architecture with the cross-cutting trustworthiness principles to operationalise.

**Figure 4:**
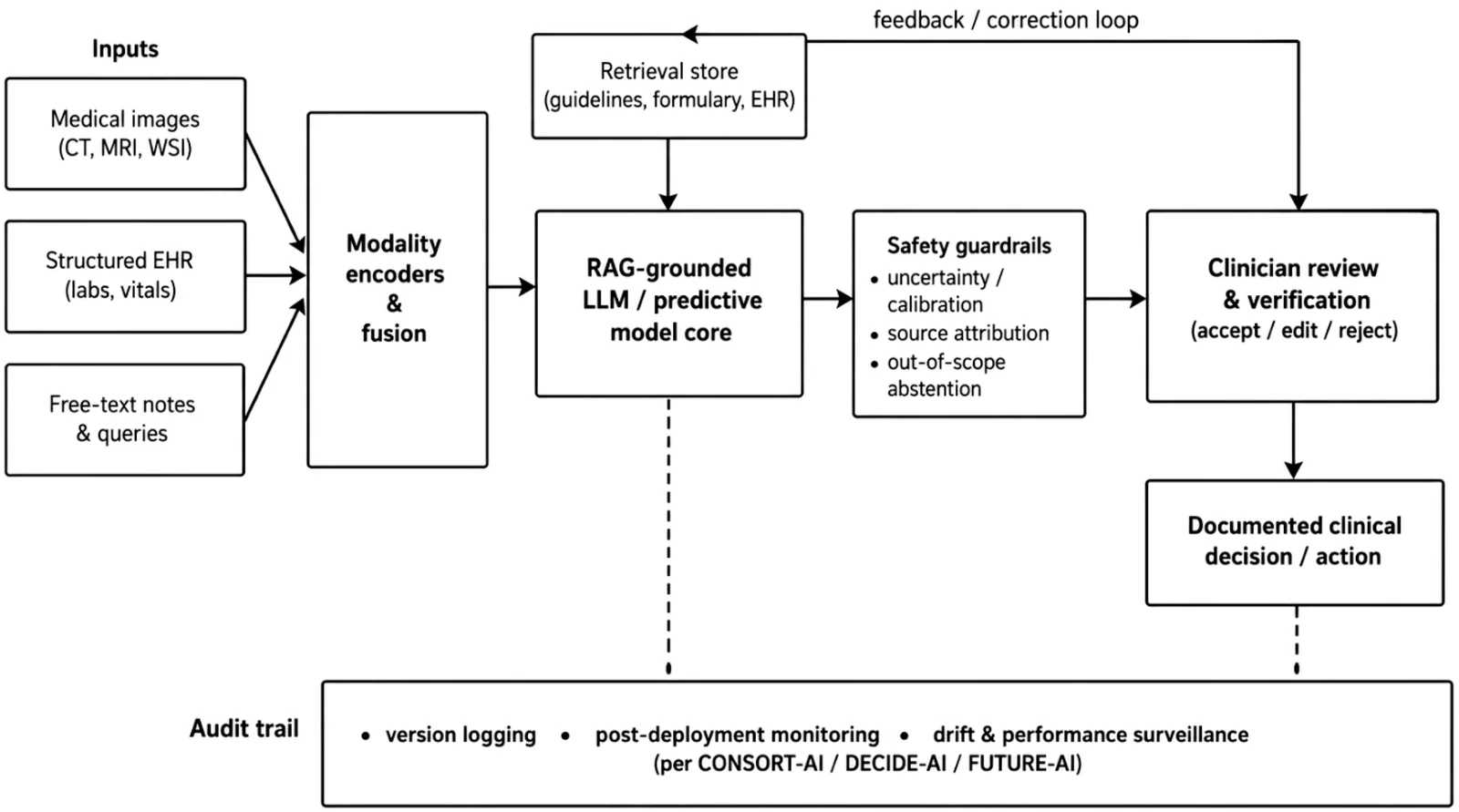
Human-in-the-loop safety architecture for a generative-AI CDSS.

In Figure 4, Multimodal inputs are encoded and fused, grounded against a retrieval store, and passed through safety guardrails (uncertainty/calibration, source attribution, out-of-scope abstention) before mandatory clinician verification. Every step is logged for audit and post-deployment monitoring, aligning the design with CONSORT-AI, DECIDE-AI and FUTURE-AI expectations. The feedback loop routes clinician corrections back to the model.

## 7 Challenges and limitations of the technology

While these developments in Sections 4–5 have been good, there are some difficulties that could arise if research and policy efforts were not targeted. Many of these challenges—including data quality, generalisability, interpretability, workflow integration and evaluation—were recognised early in foundational accounts of clinical machine learning (62) and of the specific barriers to clinical impact (63) and have not been resolved. We classify them into technical, ethical and regulatory categories.

### 7.1 Data scarcity, annotation burden and distribution shift

In the clinical settings, supervised DL is limited by labels. The cost of creating annotated medical-image datasets is high and they are geographically concentrated and subject to inter-rater variation, which introduces label noise (5, 46). Open benchmarks (MICCAI, TCIA, NIH Chestx-ray) have led to progress; however, these are too small and too limited to provide broadly generalisable models. The main challenge for multi-institutional adoption is distribution shift, which occurs when a model trained on devices, protocols, or populations that the model was not created for, is degraded when applied to these different devices, protocols, or populations (31, 46). A recent study of COVID-19 imaging machine-learning models found that of the several hundred models published, none of them were clinically useful, mainly because of the bias in the data source, methodological issues, and the lack of external validation of the models (60)—a cautionary tale about curated-data performance. Federated learning has been proposed to support training without data sharing (64) while federated training under the scenario of clinical distribution shift is still an open problem (50, 65). It has been suggested that generative AI could generate labelled data, but the usefulness of synthetic data for downstream training will depend on the quality of the generative model and models trained on small or biased datasets can reproduce or exacerbate the biases in the training data (12, 13).

### 7.2 Interpretability and the black-box problem

Diagnosis is a process which has to be accounted for: clinicians have to explain their diagnoses to patients, to fellow professionals, and to regulators. A key obstacle to uptake and accountability in deep networks, particularly those with billions of interacting parameters such as LLMs, is opacity (17, 45). This is partially addressed for CNN-based imaging using methods like *post-hoc* methods (Grad-CAM, saliency maps, SHAP) which provide limited insights into LLM decision-making (17, 49) and a major critique claim that providing *post-hoc* explanations in high-stakes settings is a false sense of security, and favors inherently interpretable models when available (66). However, other researchers have noted that explainable-AI tools are not yet clinically validated and that existing tools can be unreliable (67). Mechanistic interpretability is promising but not yet sufficiently developed and ripe for clinical use (45). Another issue of concern is calibration: LLMs are often more confident than they should be when they are incorrect, while being overly confident when they are correct can be as harmful as being underconfident when it's not (17, 24).

### 7.3 Hallucination in generative models

Hallucination—plausible-sounding but incorrect output—is the most pressing safety issue for clinical LLMs. Hallucinated clinical text (the finding of non-existent guidelines, incorrect doses and invented interactions) is difficult to identify without domain knowledge, and can be systematically dangerous (17, 20, 21). RAG can help with that by ensuring that the generation is based on retrieved facts, but it doesn't completely eliminate it, especially if the retrieved facts are incomplete, outdated, or misinterpreted by the model (16). Generative imaging is also impacted by hallucination: GANs and diffusion models can generate radiologically plausible but anatomically incorrect structures to pollute classifiers trained on synthetic data (12, 15).

### 7.4 Ethics, privacy and equity

The clinical AI training process entails privacy concerns, particularly when dealing with patient information shared between institutions or commercial services. While both HIPAA and GDPR can provide protection, both were not built with the intent of foundation-model scale data use and create potential gaps (20). Differential privacy can minimize re-identification risk at the expense of loss in accuracy (65). Another issue is equity; systems trained on data from high income, high tech environments might demonstrate systematic performance differences in low-income, low tech communities, and generative models can exacerbate this by replicating training data bias (9, 20). The risk is not theoretical, as an often-quoted study indicated that a commercial risk-prediction algorithm systematically underserved Black patients by using the cost of health care as a proxy for need (68) and classifiers based on chest radiographs have been demonstrated to under-diagnose historically under-served groups (69). The cases highlight the need to address the issue of fairness explicitly, not on the assumption that it is present.

### 7.5 Regulatory challenges

The regulation system is behind the current generative systems. The FDA's Software-as-a-Medical-Device (SaMD) guidance and the EU's AI Act are mostly based on the assumption of a “locked” algorithm; continuously updated LLMs and foundation models don't fit and performance can change between iterations (10, 20). The SaMD locked/adaptive distinction fails to adequately represent the adaptive systems that integrate external knowledge in a way that adapts to the external source. An investigation of FDA-cleared AI devices revealed that most were approved based on retrospective studies, and fewer than 1 in 5 provided multi-site or prospective evaluation and provided limited transparency on patient populations assessed (70). The number of clinical trials that have been conducted on AI-CDSS is still limited, and the regulatory science to define non-inferiority margins is in its infancy (22) – the need for which the reporting frameworks in Section 6 are meant to address.

### 7.6 Computational cost and environmental impact

The cost of training and inference of large generative models is an actual ethical and access issue. Few organisations can afford to train GPT-4 models, and for few systems have inference cost for real-time CDSS become affordable (10, 50). Until recently, the energy and carbon footprint of large-scale training has not been considered to any great degree. There are active directions that are directly relevant to equitable scaling, such as efficiency methods (distillation, quantization, mixture-of-experts) and edge inference.

### 7.7 Prospective evidence, workflow integration, clinician acceptance and deployment barriers

The biggest drawback to the current evidence base is the lack of prospective clinical trials. In the synthesis by Chen et al. (21) of 4,609 clinical-LLM records, only 19 were prospective randomised controlled trials; in the meta-analysis of predictive AI-CDSS across 50 studies and 17 specialties (22) the vast majority of evaluations were done on historical, retrospective data rather than live workflows; and in the FDA audit of AI devices, less than one in five are reported to have been evaluated across multiple sites, or prospectively evaluated (70). The evidence in the literature is largely reader-study (and benchmark) evidence, which proves that a model can execute a task, but not that it actually increases the quality of care in that task. The second barrier, somewhat independent of the first, is integration with workflow. The benefit of an accurate model will be realized only if the output of the model is accepted by the right clinician at the right time, in the right format, in the right place, and at the right time. In reality, this translates to integration with the EHR and PACS, latency sufficiently low to not disrupt the task and alert thresholds set such that false-positives are not an undue burden (which is known as ‘alarm fatigue' and is a documented source of the prospective–retrospective performance gap (22, 45).

Ignored systems, no matter how good their AUROC, are not going to improve outcomes. The third barrier is clinician acceptance and cannot be broken down into accuracy. DL-based CDSS have proven to be more discriminative than rule-based systems but less accepted due to not being able to explain its reasoning (45). Two types of failure are also salient in shaping the acceptance: under-reliance, in which the clinician dismisses advice that is correct, and automation bias, in which the clinician takes advice that is incorrect without questioning it, documented in primary care with generative AI, where passive uptake of the AI output may lead to systematic error (11). Unresolved liability: When a clinician implements or dismisses an AI recommendation that fails, it is unclear who is responsible, making this another obstacle to uptake (20). These challenges are compounded by regulatory requirements. The locked/adaptive distinction does not cover a system whose knowledge is updated at inference time, as with LLMs and RAGs, which change their behaviour as their models and retrieval corpora are updated (10, 20). There are no published non-inferiority margins for clinical AI performance (22).

Practical deployment barriers include the following: the cost to perform inference with a real-time LLM-CDSS could be too large for a resource-constrained provider (10, 50); even tools that have been cleared will require local validation which may not be available in institutions where it is needed; and model drift monitoring after deployment is not done with resources. These are all together responsible for the fact that so few systems that work well in assessment are widely used in clinical practice and will form the agenda of Section 8.

## 8 Future research directions

The problems outlined above form a research program. On the journey from proof-of-concept to safe, fair and trusted clinical AI, we find six directions to highlight.

### 8.1 Explainable and trustworthy AI

Going beyond *post hoc* explanations of saliency into explanations aligned with clinical reasoning, such as differential diagnosis lists and weighed evidence beyond mere attribution at the level of pixels or tokens (17, 45). Uncertainty quantification methods (Bayesian deep learning, conformal prediction, temperature scaling) should be standard and enable clinicians to act on calibrated outputs (24) following the FUTURE-AI explainability and robustness principles (71).

### 8.2 Multimodal integration

Imaging combined with clinical text and structured EHR and genomics is likely to be most impactful (31, 50). A promising one is to use biomedical foundation models that are pretrained on multiple modalities, such as contrastive encoding of images and text using CLIP-style models. Great efforts are still needed for future research to focus on efficient cross modal attention and modality-agnostic pretraining and evaluation metrics that reflect integrated system performance (8, 50).

### 8.3 Federated and privacy-preserving learning

Federated learning is a method for multi-site training and overcoming privacy and single-site distribution shift (46, 65). Pockets include federated optimisation that is immune to multi-site drift, communication-efficient updates, and differential privacy with little loss of performance; secure and privacy-preserving methods for medical imaging (encrypted computation, secure aggregation and differential privacy) complement each other, and have been surveyed (72). Traceable consent and attribution can be provided through provenances at the site level (20, 50).

### 8.4 Human-in-the-loop validation

To move from retrospective benchmarking to prospective deployment, validated human-in-the-loop models need to be defined for scope, presentation, and verification (Figure 4) (17, 22). Focus should be on trial designs with measures of clinician–AI team performance rather than performance of AI only, and on understanding of automation bias, alarm fatigue, and skill decay; and on adaptive allocation of tasks to scale oversight to complexity, model confidence, and risk – reported per DECIDE-AI and, at trial stage, CONSORT-AI (73, 74).

### 8.5 Efficient and lightweight architectures

Architectures that are close to the state-of-the-art under stringent compute requirements are needed for cost-effective deployment in community hospitals, primary care and low-resource settings. Key techniques: distillation, pruning, quantization aware training and mixture of experts; on edge deployment (e.g., portable ultrasound) is an important equity use case.

### 8.6 Prospective trials and regulatory science

Randomised trials (or, when randomisation is unfeasible, robust, well-designed observational studies) with pre-specified end points are required to assess AI-CDSS against actual end points, not proxies (22, 28). Regulatory science needs to establish standards for adaptive and generative systems, not simply the frozen-algorithm type, and a series of consortia involving vendors, providers, regulators and patient advocates to help develop the evidence base for safe and equitable deployment.

### 8.7 A staged roadmap for research and practice

The six directions above are not equally important; they are not independent; and, they are of practical use when sequenced. Table 6 thus delineates a road-mapping progression between what is actionable today, what needs to be sustained as a programmatic investment and who is responsible and what would be considered success at each stage. Organising principle: Validation, governance and human-AI interaction is the limiting factor for clinical impact rather than capability of the model, which will continue to be provided by commercial development as long as academic efforts exist.

**Table 6:** Staged research and implementation roadmap for deep learning and generative AI in medical imaging and CDSS.

Horizon	Priority action	Primary stakeholders	Success criterion
Near term (0–2 years)	Report all new imaging and CDSS models according to CLAIM and TRIPOD+AI guidelines; publish external-validation results and calibration measures in addition to discrimination metrics; prospectively register clinical AI studies.	Researchers, journals, reviewers	Published models are transparently reported, independently appraisable, and their generalisability can be verified by external researchers.
Near term (0–2 years)	Deploy LLM-based CDSS only within the human-in-the-loop safety architecture (Figure 4), incorporating defined scope, mandatory clinician verification, source attribution, abstention mechanisms, and audit logging.	Healthcare systems, AI vendors	No LLM-generated recommendation reaches a patient without documented clinician verification.
Medium term (2–5 years)	Conduct DECIDE-AI compliant early clinical evaluations followed by CONSORT-AI and SPIRIT-AI randomized clinical trials, evaluating clinician–AI team performance and patient outcomes rather than model accuracy alone.	Clinical investigators, funding agencies	Robust prospective clinical outcome evidence is available for high-volume CDSS applications.
Medium term (2–5 years)	Establish federated, privacy-preserving, multi-centre validation infrastructures and perform routine fairness auditing across demographic and clinical subgroups.	Research consortia, regulatory agencies	Performance, robustness, and fairness are demonstrated across institutions and patient populations before deployment.
Medium term (2–5 years)	Develop calibration and uncertainty estimation as standard model outputs (e.g., conformal prediction, temperature scaling), together with clinically meaningful explanations rather than only saliency maps.	Researchers	Clinicians receive reliable confidence estimates and understand when AI recommendations should or should not be trusted.
Long term (5+ years)	Develop regulatory science for adaptive and generative AI systems, including non-inferiority margins, post-deployment drift monitoring, and approval pathways that support continuously learning models.	Regulatory agencies, AI vendors, academia	Generative AI-based CDSS can be approved, monitored, and safely updated within an established regulatory framework.
Long term (5+ years)	Develop efficient, lightweight, and edge-deployable AI models to extend clinically validated AI systems to community healthcare, primary care, and resource-limited settings.	Researchers, global health organizations, funding agencies	The benefits of clinically validated AI become accessible beyond tertiary care centres and high-resource healthcare systems.

## 9 Discussion

There is sound evidence for a measured assessment: not one that is dismissive or promotional. Three themes of analysis emerge: a maturity gradient among AI systems, a capability–reliability trade-off, and a co-evolution of technology and institutional, ethical and regulatory frameworks.

### 9.1 Maturity gradient: from deployed to emerging

Evidence we have is organized by maturity. Supervised imaging DL (CNNs and U-Net variants) is the most developed: it is incorporated into commercial radiology products, approved for use in a few jurisdictions for specific indications, and is being tested in prospective studies (1, 4, 22). It has fairly good characteristics and limitations, as a result of consistent investment, ample labelled data, and repeated translation. It would be premature to drive the statement that the evidence suggests AI in imaging is generally “clinically deployed” as there is no universal agreement on what this means. Readiness is specific to the task, jurisdiction and workflow, and even established tools need local validation. The newer generation of ViTs and LLMs are in a promising class that performs well on benchmarks but lacks clinical validation studies (8, 26, 50). LLM-based CDSSs are the least developed: transforming in terms of capability but not reliable or safe or even recognized as a CDSS by regulators yet (17, 21, 23). This maturity gradient aligns with early influential descriptions of the merging of human and artificial intelligence in medicine, which predicted that the critical challenges would involve not only algorithmic, but also human-factors, and evidence-based issues (75). The research implication is that it is not simply more powerful models that need to be created, commercial incentives will provide these, it is the validation, governance and human–AI interaction processes that are necessary to deploy the models safely that need to be developed.

### 9.2 Capability–reliability trade-off

The trend in Table 4 and Figure 3 is consistent: the more capable a system becomes — reasoning over more varied data types and producing more flexible recommendations — the less reliable, interpretable and controllable it becomes. This is not only an artifact of engineering to solve but a portion of probabilistic models that sample from learned distributions instead of deterministic logic. The result is that capability and reliability need to be measured by a task. In situations requiring action before a decision (such as sepsis management, stroke triage, ICU dosing), the use of validated predictive DL-CDSS might be more suitable than flexible LLMs. LLM reasoning could be appropriate for tasks with lower clinical stakes (first differential list, patient information, documentation), where the clinician must review the output (18, 19, 25).

### 9.3 Traditional ML, DL and generative AI: a contextual comparison

These families represent an expansion of the clinical AI toolkit rather than a linear progression in which each supersedes the last. In high stakes classification tasks with limited data availability and high interpretability requirements, e.g., EHR risk calculators and eligibility prediction from structured data, traditional ML (logistic regression, random forests, SVMs) is still useful (45). For perceptual tasks (imaging, speech, ECG), DL is preferred (1, 3). While the clinical use of AI has been enhanced by LLMs' ability to generate text, infer, and interact with natural language, the downside is that they lack reliability and interpretability (9, 17). The most effective deployed solutions are likely to combine all three — traditional ML for structured risk prediction, DL for image analysis, and LLMs for language tasks with each component confined to its validated operating range and subject to human oversight.

### 9.4 The translational gap

One of the most frequently observed findings across the studies reviewed is that there is a discrepancy in the results of the algorithms in curated retrospective data and clinical outcomes when applied to prospective data. This has been made quite clear by the meta-analysis in (22) which, across 50 studies and 17 specialties, showed pooled discrimination of only moderate quality, and more importantly, most of the evaluations were based on historical data rather than using real, live clinical workflows, making reported accuracy very limited predictors of benefit. This is reflected by the results of the LLM-assisted synthesis in (21) which identified 1,857 simulated and 1,704 examination-style studies, along with 1,048 real-world clinical-LLM studies published from January 2022 to September 2025, of which only 19 were prospective randomised trials. The causes—distribution drift, workflow disruption, alarm fatigue, low clinician adherence and limited implementation-science support—are systemic. To close this gap, a move from algorithm-centred questions (how good is the model on the benchmark?) to system-centred questions (how good is the clinician–AI system for patients?), and hence study design and funding and incentives, will be required (22, 28).

### 9.5 Does current evidence support routine deployment of LLM-powered CDSS?

Evidence reviewed in this question does not support the routine independent use of CDSS powered by LLM. They must stay an assistant, under explicit and strict human control. There are four lines of evidence that lead to this conclusion. First, the potential evidence base is very small: 19 out of 4,609 clinical-LLM records screened for randomised trials (21) and essentially no outcome-level data. Second, at sites where frontier LLMs have been evaluated against real clinical data (not examinations-like benchmarks), they have been found to have failed safety-relevant tasks: Hager et al. (17) tested 2,400 real intensive-care cases and found that models did not follow diagnostic guidelines, misinterpreted laboratory results, and were sensitive to the order and quantity of information presented, prompting the authors explicitly to conclude that current LLMs are not ready for autonomous clinical decision-making.

Third, head-to-head evidence supports augmentation over substitution: the configuration of the pharmacist-plus-LLM was approximately 32% more successful than the configuration of pharmacist alone in detecting drug-related problems (18), and in gastroenterology, recommendations made by the LLM did not reach trainee level but were lower than those made by the subspecialist (19). Fourth, the failure modes are non-accidental and not a consequence of probabilistic generation at the token level (Section 9.2); hallucination, miscalibration and opacity are not outcomes of RAG, but rather are failure modes that are exacerbated by it (16). This decision is not generalizable to all tasks and the question of interest is not if LLM-CDSS are solved, but which task, under which supervision. When the clinical situation is low stakes, the output is easily verifiable with a single glance, and a clinician reviews it before it is presented to the patient — documentation alone, patient-facing explanation alone, generating a first differential list for a clinician to eliminate the evidence is adequate to support supervised use. If an error cannot be easily caught or if the error is made on the patient without a human judgement — autonomous dosing, unreviewed triage, direct-to-patient advice — it isn't.

The minimal requirements for any deployment are thus: defined and validated scope of use; mandatory clinician verification prior to influencing care; source attribution and abstention when out of scope; full audit logging; and prospective monitoring (i.e., the safety architecture in Figure 4, as assessed in DECIDE-AI and, at trial stage, CONSORT-AI (73, 74).

## 10 Limitations of this review

A few caveats must be noted. First, it is a structured critical narrative review rather than a systematic review – the focus of 37 studies (from a total of 80 studies) has been selected so as to highlight the development of the field and is not exhaustive, so that a relevant primary study and review is inevitably not included. Second, not registered and not carried out systematically, therefore it is not reproducible; publication bias towards positive results possible and over-representation of reviews versus primary evidence. Thirdly, only English-language, peer-reviewed sources are used, which means that non-English and grey literature may not be relevant. Fourth, individual studies were not assessed with a formal risk of bias instrument (PROBAST + AI (76) but an appraisal is provided descriptively in Supplementary Table S1. Fifth, the field is dynamic and some information on 2025–2026 may be replaced by the time of publication. Any reader should take note of the conclusions and we see conversion to registered systematic reviews with formal appraisal as the natural next step.

## 11 Conclusion

A comprehensive evidence base was derived from 80 sources, including 37 key studies, 43 landmark primary studies, foundational papers, and reporting standards for clinical-AI, which were assessed using a common analytical framework that ranged from DL and generative AI in medical imaging to CDSS in the field of clinical decision support. There are four conclusions, each based on evidence. First, supervised DL (CNNs, U-net variants, and more recently ViTs) can provide clinically useful accuracy on well-scoped imaging tasks, but only after local validation, not across-the-board as “deployment-ready.” Second, the default approach to supervised DL is the U-net, which is by far the most common. Third, generative imaging (GANs, diffusion) can alleviate data scarcity by creating synthetic data and cross-modal translation; current hallucination risk and lack of correlation between synthetic image metrics and clinical value prevent it from being a tool for clinical use. Fourth, LLM and RAG based CDSS have very real potential for guideline-concordant reasoning, medication-safety checking and differential diagnosis – but current governance structure is still insufficient, and mandatory clinician review, full audit trails and human-in-the-loop evaluation is necessary for deployment; these are currently at the feasibility-study stage, with diabetes and gastroenterology and internal medicine showing early signs of piloting. Safe deployment is the key constraint on clinical impact, in our assessment as an interpretation of the evidence and not as a fact, as it requires the validation and governance of clinical AI systems, as well as human–AI protocols and reporting standards (Section 6) necessary for safe, fair, explainable and accountable systems. This will need to be a transdisciplinary effort involving coordinated research, practice, regulation, ethics and patient advocacy in AI.

### The strengths of this review

Four of the insights are new in comparison to the existing surveys. First, while almost always reviewed separately, imaging AI and CDSS can be examined on a common evidence landscape, judged by a single, nine-dimensional rubric, and linked to a capability–reliability trade-off that is not apparent in viewing each component individually alone — the most functional (LLM-CDSS) are systematically the least interpretable, calibrated, and validated; that is not a temporary engineering problem, but a structural property of probabilistic generation. Second, the maturity gradient we have developed points to a shift in the binding constraint to clinical impact: it is no longer model capability, which commercial development is able to provide, but validation, governance and human–AI interaction protocols, which it can't. Third, we present a quality appraisal for each study included in the review, instead of reporting simple performance metrics, to demonstrate that the apparent performance of clinical AI is overwhelmingly retrospective and benchmark evidence, not algorithmic deficits, that is why there is a translation gap. Fourth, we map that diagnosis to the clinical-AI reporting-standards landscape, thereby turning the diagnosis into a prescription: a staged evidence pathway from CLAIM/TRIPOD + AI-compliant technology development reporting; to DECIDE-AI early, clinical evaluation; to CONSORT-AI trials (Sections 6 and 8.7). This synthesis is sufficient to propose a conclusion that should not be taken as a final truth: LLM-powered CDSS are not suitable for automatic use, and should be used in an assisted manner where they are used under strict human control (Section 9.5).

## Supplementary material

Supplementary Data Sheet 1 Evidence-and-appraisal matrix for the 80 included sources: study type, validation design, dataset scope, external-validation status and indicative clinical-readiness tag.

Supplementary Data 2 Full Boolean search strings for PubMed/MEDLINE, IEEE Xplore, Web of Science and Scopus (final search 30 June 2026).
