# Supplementary material for: Deep learning and generative AI for medical imaging and clinical decision support systems: a structured critical review
Source: Front Digit Health. 2026 Aug 18;8:1890072. doi: 10.3389/fdgth.2026.1890072 (PMC13530434; doi:10.3389/fdgth.2026.1890072)
Supplement: Supplementary file 1 [file Datasheet1.pdf]

Supplementary Table S1 — Per-study evidence-quality appraisal

"Deep Learning and Generative AI for Medical Imaging and Clinical Decision Support Systems: A Structured Critical Review"

Appraisal of all 80 references: the 37 core included studies ([1]–[37]) and the 43 supplementary references ([38]–[80]). "Not reported" (NR) = field not stated in the source; "N/A" = field not applicable to a secondary/review or opinion article. Risk-of-bias and evidence-strength ratings are qualitative descriptive judgements informed by study design, not a formal scored instrument; a PROBAST+AI appraisal is recommended for a future systematic-review version. Rows [38]–[80] are landmark studies, foundational works and reporting guidelines.

Ref	Year	Study design	Sample size / scope	Validation strategy	External val.	Prospective val.	Risk of bias	Evidence strength	Clinical readiness	Major limitation
1	2017	Narrative review	N/A (review)	N/A (secondary)	N/A	N/A	High (narrative)	Low (secondary)	Foundational	No primary data; non-systematic selection
2	2021	Review	N/A (review)	N/A (secondary)	N/A	N/A	Moderate	Low–Mod (secondary)	Established	Breadth over depth; limited appraisal
3	2017	Review	N/A (review)	N/A (secondary)	N/A	N/A	High (narrative)	Low (secondary)	Foundational	Early scope; now partly dated
4	2020	Review	N/A (review)	N/A (secondary)	N/A	N/A	Moderate	Low–Mod (secondary)	Workflow-oriented	Descriptive; little quantitative synthesis
5	2023	Benchmarking review	Multiple public datasets	Retrospective / benchmark	Limited	No	Moderate	Moderate (benchmark)	Benchmark	Benchmark-bound; curated data
6	2021	Tutorial / review	N/A (review)	N/A (secondary)	N/A	N/A	High (narrative)	Low (secondary)	Educational	Instructional; not evaluative
7	2022	Review (preprint)	N/A (review)	N/A (secondary)	N/A	N/A	High (preprint)	Low (secondary)	Emerging	Preprint; not peer-reviewed
8	2020	Review	N/A (review)	Retrospective (surveyed)	N/A	N/A	Moderate	Moderate (secondary)	Established	Segmentation-focused; label-noise emphasis
9	2021	Review	N/A (review)	N/A (secondary)	N/A	N/A	Moderate	Low–Mod (secondary)	Established	Descriptive taxonomy; limited appraisal
10	2019	Systematic review	N/A (review)	Secondary synthesis	N/A	N/A	Moderate	Moderate (secondary)	Established	Pre-2019 scope; GAN field since evolved
11	2023	Empirical comparison	Multiple GAN variants	Retrospective / benchmark	Limited	No	Moderate	Moderate (benchmark)	Benchmark	Metric–reader disagreement; no clinical endpoint
12	2022	Primary (method + reader)	Single / limited cohort	Retrospective + reader study	No	No	Moderate–High	Low–Moderate	Reader-validated	Small single set; reader study only
13	2021	Primer / review	N/A (review)	N/A (secondary)	N/A	N/A	High (narrative)	Low (secondary)	Educational	Primer; not evaluative
14	2017	Review	N/A (review)	Retrospective (surveyed)	N/A	N/A	Moderate	Low–Mod (secondary)	Established	Broad healthcare scope; shallow per-topic
15	2023	Review (preprint)	N/A (review)	N/A (secondary)	N/A	N/A	High (preprint)	Low (secondary)	Emerging	Preprint; not peer-reviewed
16	2023	Review	N/A (review)	N/A (secondary)	N/A	N/A	Moderate	Low (secondary)	Emerging	Descriptive; limited critical depth
17	2025	Review	N/A (review)	N/A (secondary)	N/A	N/A	Moderate	Low–Mod (secondary)	Emerging	Rapid-field snapshot; may date quickly
18	2025	Survey (foundation models)	N/A (review)	N/A (secondary)	N/A	N/A	Moderate	Low–Mod (secondary)	Emerging	Survey; few clinical outcomes
19	2024	Scoping review	N/A (review)	Secondary synthesis	N/A	N/A	Moderate	Moderate (secondary)	Emerging	Scoping; heterogeneity of included work
20	2025	Governance review	N/A (review)	N/A (secondary)	N/A	N/A	Moderate	Low–Mod (secondary)	Governance	Policy focus; limited empirical data
21	2024	Review	N/A (review)	N/A (secondary)	N/A	N/A	Moderate	Low (secondary)	Emerging	Primary-care focus; anecdotal examples

Ref	Year	Study design	Sample size / scope	Validation strategy	External val.	Prospective val.	Risk of bias	Evidence strength	Clinical readiness	Major limitation
22	2025	Ethical framework / opinion	N/A (opinion)	N/A	N/A	N/A	High (opinion)	Low (opinion)	Governance	Opinion; not evidence-based
23	2025	Scoping review	N/A (review)	Secondary synthesis	N/A	N/A	Moderate	Moderate (secondary)	Emerging	Scoping; medication-safety subset only
24	2025	Review	Surveyed benchmarks	Secondary (benchmarks)	N/A	N/A	Moderate	Moderate (secondary)	Emerging	Benchmark-derived; mixed reasoning results
25	2025	Systematic review	87 studies synthesised	Secondary synthesis	N/A	N/A	Low–Moderate	Moderate–High (secondary)	Methodological	Reporting heterogeneity across included studies
26	2026	Benchmarking	Public Q&A datasets	Benchmark (Q&A)	Limited	No	Moderate	Moderate (benchmark)	Benchmark	Q&A proxy; not bedside reasoning
27	2024	Primary (RAG evaluation)	Simulated vignettes	Simulated vignettes	No	No (simulated)	Moderate	Low–Moderate	Pilot / simulation	Simulated, not real patients
28	2024	Primary (empirical, MIMIC)	2,400 real cases (4 pathologies)	Retrospective real-case	No	No	Low–Moderate	Moderate	Methodological	USA-centric data; abdominal cases only
29	2026	LLM-assisted review / meta	4,609 studies screened	Secondary synthesis	N/A	N/A	Low–Moderate	Moderate–High (secondary)	Methodological	LLM-assisted screening; residual extraction error
30	2025	Systematic review	Surveyed HC-LLM apps	Secondary synthesis	N/A	N/A	Low–Moderate	Moderate (secondary)	Methodological	Few evaluations used real patient care
31	2025	Perspective / design	N/A (perspective)	N/A	N/A	N/A	High (perspective)	Low (opinion)	Conceptual	Conceptual; no evaluation
32	2025	Primary (comparative)	Real internal-med cases (small)	Retrospective real-case	No	No	Moderate	Moderate	Pilot	Single-centre; small sample
33	2025	Primary (prospective cross-over)	91 scenarios / 40 vignettes, 16 specialties	Prospective cross-over	No	Yes	Low–Moderate	Moderate–High	Pilot (augmentation)	Simulated prescribing, not live orders
34	2025	Primary (evaluation)	NR (specialty cases)	Structured-input evaluation	No	No	Moderate	Moderate	Pilot	Below subspecialist; input-dependent
35	2024	Review	N/A (review)	N/A (secondary)	N/A	N/A	Moderate	Low–Mod (secondary)	Mixed	CDSS taxonomy; adoption barriers noted
36	2026	Systematic review + meta-analysis	50 studies / 17 specialties	Pooled (mostly retrospective)	Pooled/heterogeneous	Few prospective	Moderate	Moderate–High (secondary)	Deployed (select centres)	Most primary studies retrospective; heterogeneity
37	2025	Primary (prospective)	Clinical cohort (NR size)	Prospective	NR	Yes	Moderate	Moderate	Prospective utility	Limited reporting; single domain (diabetes)
38	2020	Reporting guideline (CONSORT-AI)	N/A (guideline)	N/A	N/A	N/A	N/A	High (consensus standard)	Reporting standard	Scope limited to AI clinical trials
39	2020	Reporting guideline (SPIRIT-AI)	N/A (guideline)	N/A	N/A	N/A	N/A	High (consensus standard)	Reporting standard	Trial protocols only
40	2024	Reporting guideline (TRIPOD+AI)	N/A (guideline)	N/A	N/A	N/A	N/A	High (consensus standard)	Reporting standard	Prediction-model studies
41	2020	Reporting checklist (CLAIM)	N/A (guideline)	N/A	N/A	N/A	N/A	High (consensus standard)	Reporting standard	Imaging AI only
42	2022	Reporting guideline (DECIDE-AI)	N/A (guideline)	N/A	N/A	N/A	N/A	High (consensus standard)	Reporting standard	Early live-clinical evaluation stage
43	2021	Guideline protocol (STARD-AI)	N/A (protocol)	N/A	N/A	N/A	N/A	Moderate (protocol)	Protocol	Full guideline not yet finalised
44	2025	Risk-of-bias tool (PROBAST+AI)	N/A (tool)	N/A	N/A	N/A	N/A	High (consensus tool)	Appraisal tool	Prediction models only
45	2020	Systematic review (meta-research)	Surveyed DL-vs-clinician studies	Secondary synthesis	N/A	N/A	Low–Moderate	Moderate–High (secondary)	Methodological	Included studies poorly reported

Ref	Year	Study design	Sample size / scope	Validation strategy	External val.	Prospective val.	Risk of bias	Evidence strength	Clinical readiness	Major limitation
46	2025	Consensus framework (FUTURE-AI)	N/A (framework)	N/A	N/A	N/A	N/A	High (consensus framework)	Framework	Guiding principles, not a checklist
47	2015	Foundational review	N/A (review)	N/A	N/A	N/A	N/A	High (landmark)	Foundational	Not medical-specific
48	2015	Primary (segmentation method)	Biomedical benchmark sets (small)	Benchmark	No	No	Moderate	High (landmark method)	Established method	Small original datasets
49	2017	Survey / review	N/A (review)	N/A	N/A	N/A	N/A	Moderate	Foundational	Pre-2017 scope
50	2017	Primary (diagnostic classification)	Large dermatology image set	Retrospective + reader study	No	No	Moderate	Moderate–High	Reader-validated	Single-modality; retrospective
51	2016	Primary (diagnostic screening)	Large retinal-image set	Retrospective + graded reference	Yes (2 validation sets)	No	Low–Moderate	High	Validated (retrospective)	Retrospective; grader-defined reference
52	2020	Primary (cancer screening)	Multi-site screening cohorts (US/UK)	Retrospective + reader study	Yes (cross-population)	No	Moderate	Moderate–High	Reader-validated	Retrospective; generalisation debated
53	2019	Primary (cancer screening)	Large lung-CT cohort	Retrospective	Limited	No	Moderate	Moderate	Retrospective	Single-dataset; not prospective
54	2021	Primary (segmentation method)	23 public segmentation tasks	Cross-task benchmark	Yes (cross-dataset)	No	Low–Moderate	High (method)	Established method	Benchmark, not clinical outcome
55	2017	Primary (architecture method)	NLP benchmarks	Benchmark	N/A	N/A	Moderate	High (landmark)	Foundational method	Not medical
56	2021	Primary (architecture method)	Large image benchmarks	Benchmark	N/A	N/A	Moderate	High (method)	Foundational method	Data-hungry; large-scale pretraining
57	2021	Primary (segmentation method)	Segmentation benchmarks	Benchmark	No	No	Moderate	Moderate–High	Method	Benchmark only
58	2014	Primary (generative method)	Image benchmarks	Benchmark	N/A	N/A	Moderate	High (landmark)	Foundational method	Not medical; training instability
59	2020	Primary (generative method)	Image benchmarks	Benchmark	N/A	N/A	Moderate	High (method)	Foundational method	Compute-intensive sampling
60	2022	Primary (generative method)	Image benchmarks	Benchmark	N/A	N/A	Moderate	High (method)	Foundational method	General-domain, not clinical
61	2023	Survey / review	N/A (review)	N/A	N/A	N/A	N/A	Moderate	Foundational	Survey of medical diffusion
62	2023	Perspective	N/A (perspective)	N/A	N/A	N/A	N/A	Moderate (opinion)	Conceptual	Vision paper, not an evaluation
63	2023	Primary (LLM benchmark)	Medical QA benchmarks	Benchmark + human evaluation	No	No	Moderate	Moderate–High	Benchmark	Exam-style, not bedside outcomes
64	2023	Primary (LLM benchmark, preprint)	Medical QA benchmarks	Benchmark + human evaluation	No	No	Moderate–High (preprint)	Moderate	Benchmark	Preprint; exam-style
65	2023	Review	N/A (review)	N/A	N/A	N/A	N/A	Moderate	Emerging	Rapid-field snapshot
66	2023	Primary (imaging foundation model)	Millions of retinal images (self-supervised)	Retrospective, multi-task	Yes (multi-cohort)	No	Low–Moderate	Moderate–High	Emerging	Retina-specific; prospective value pending
67	2024	Primary (segmentation foundation model)	Large multi-modality image corpus	Cross-modality benchmark	Partial	No	Moderate	Moderate–High	Emerging	Uneven on specialised modalities
68	2023	Primary (segmentation method)	Very large (SA-1B: ~11M images)	Benchmark	N/A	N/A	Low–Moderate	High (method)	Foundational method	General-domain; not medical
69	2020	Primary (retrieval method)	NLP knowledge-intensive tasks	Benchmark	N/A	N/A	Moderate	High (method)	Foundational method	Not clinical
70	2019	Perspective / review	N/A (review)	N/A	N/A	N/A	N/A	Moderate	Foundational	Landmark opinion piece

Ref	Year	Study design	Sample size / scope	Validation strategy	External val.	Prospective val.	Risk of bias	Evidence strength	Clinical readiness	Major limitation
71	2019	Review	N/A (review)	N/A	N/A	N/A	N/A	Moderate	Foundational	General overview
72	2019	Analysis / perspective	N/A (perspective)	N/A	N/A	N/A	N/A	Moderate	Conceptual	Barriers analysis, not primary data
73	2021	Analysis (regulatory)	FDA-cleared AI/ML devices	Document analysis	N/A	N/A	Low–Moderate	Moderate	Regulatory evidence	Most clearances retrospective; limited transparency
74	2021	Systematic review	300+ COVID-19 ML models	Secondary methodological appraisal	N/A	N/A	Low	Moderate–High (secondary)	Methodological	None found clinically usable
75	2019	Primary (algorithmic-bias study)	Large health-system cohort	Retrospective	No	No	Low–Moderate	High	Landmark (bias)	Single US health system
76	2021	Primary (fairness audit)	Large chest-radiograph datasets	Retrospective, multi-dataset	Yes (cross-dataset)	No	Low–Moderate	Moderate–High	Landmark (equity)	Retrospective
77	2019	Perspective	N/A (perspective)	N/A	N/A	N/A	N/A	Moderate	Conceptual	Argument, not an evaluation
78	2021	Review / perspective	N/A (review)	N/A	N/A	N/A	N/A	Moderate	Conceptual	Explainability critique
79	2020	Review	N/A (review)	N/A	N/A	N/A	N/A	Moderate	Emerging	Federated-learning overview
80	2020	Review	N/A (review)	N/A	N/A	N/A	N/A	Moderate	Emerging	Privacy-methods overview
