# Supplementary material for: Deep learning and generative AI for medical imaging and clinical decision support systems: a structured critical review
Source: Front Digit Health. 2026 Aug 18;8:1890072. doi: 10.3389/fdgth.2026.1890072 (PMC13530434; doi:10.3389/fdgth.2026.1890072)
Supplement: Supplementary file 2 [file Datasheet2.pdf]

Supplementary Material S2

Full database search strings

Deep learning and generative AI for medical imaging and clinical decision support systems: a structured critical review

1. Overview

This file provides the complete, platform-specific Boolean search strings referenced in Section 2.2 of the main text. Candidate literature was retrieved from four bibliographic databases — PubMed/MEDLINE, IEEE Xplore, Web of Science Core Collection and Scopus — and supplemented by citation chasing of the reference lists of pertinent reviews.

- Final search date: 30 June 2026.
- Language limit: English.
- Publication-date range searched: 2014–2026.
- Records from all sources were pooled, de-duplicated, then screened by title/abstract and at full text against the eligibility criteria in Section 2.3.

Note: the same three-concept Boolean logic was applied across all databases; only the field tags and syntax differ per platform, so each string below is the platform-adapted equivalent of the PubMed/MEDLINE base string.

2. Search concepts

The query combines three concept blocks with the Boolean operator AND; terms within each block are combined with OR.

- Block A — technology / architecture: "deep learning", "convolutional neural network", "U-Net", "vision transformer", "generative adversarial network", "diffusion model", "large language model", "retrieval-augmented generation".
- Block B — application / clinical domain: "medical imaging", radiology, pathology, segmentation, "clinical decision support", CDSS, "medication safety".
- Block C — clinical context: clinical, medical, health.

3. PubMed/MEDLINE

3.1 Base string (as reported in Section 2.2):

```
("deep learning" OR "convolutional neural network" OR "U-Net" OR "vision transformer" OR "generative adversarial network" OR "diffusion model" OR "large language model" OR "retrieval-augmented generation") AND ("medical imaging" OR radiology OR pathology OR segmentation OR "clinical decision support" OR CDSS OR "medication safety") AND (clinical OR medical OR health)
```

3.2 Reproducible field-tagged version (title/abstract, with limits applied):

```
(("deep learning"[tiab] OR "convolutional neural network"[tiab] OR "U-Net"[tiab]
  OR "vision transformer"[tiab] OR "generative adversarial network"[tiab]
  OR "diffusion model"[tiab] OR "large language model"[tiab]
  OR "retrieval-augmented generation"[tiab])
AND ("medical imaging"[tiab] OR radiology[tiab] OR pathology[tiab]
  OR segmentation[tiab] OR "clinical decision support"[tiab] OR CDSS[tiab]
  OR "medication safety"[tiab])
AND (clinical[tiab] OR medical[tiab] OR health[tiab]))
Filters: English[lang]; 2014:2026[dp]
```

4. IEEE Xplore (Command Search)

Run in the IEEE Xplore "Command Search" interface; the "All Metadata" field is used to approximate title/abstract/keyword coverage. Year and language are applied as post-search facet filters.

```
(("All Metadata":"deep learning" OR "All Metadata":"convolutional neural network"
  OR "All Metadata":"U-Net" OR "All Metadata":"vision transformer"
```

```
  OR "All Metadata":"generative adversarial network"
  OR "All Metadata":"diffusion model" OR "All Metadata":"large language model"
  OR "All Metadata":"retrieval-augmented generation")
AND ("All Metadata":"medical imaging" OR "All Metadata":radiology
  OR "All Metadata":pathology OR "All Metadata":segmentation
  OR "All Metadata":"clinical decision support" OR "All Metadata":CDSS
  OR "All Metadata":"medication safety")
AND ("All Metadata":clinical OR "All Metadata":medical OR "All Metadata":health))
Filters (facets): Year 2014-2026; language English
```

5. Web of Science Core Collection

Topic search (field tag TS=) covers title, abstract, author keywords and Keywords Plus. Language and publication-year limits applied as refinements.

```
TS=(("deep learning" OR "convolutional neural network" OR "U-Net"
  OR "vision transformer" OR "generative adversarial network"
  OR "diffusion model" OR "large language model"
  OR "retrieval-augmented generation")
AND ("medical imaging" OR radiology OR pathology OR segmentation
  OR "clinical decision support" OR CDSS OR "medication safety")
AND (clinical OR medical OR health))
Refined by: Languages = English; Publication Years = 2014-2026
Edition: Web of Science Core Collection
```

6. Scopus

TITLE-ABS-KEY searches article title, abstract and keywords; language and year limits are embedded in the query.

```
TITLE-ABS-KEY(("deep learning" OR "convolutional neural network" OR "U-Net"
  OR "vision transformer" OR "generative adversarial network"
  OR "diffusion model" OR "large language model"
  OR "retrieval-augmented generation")
AND ("medical imaging" OR radiology OR pathology OR segmentation
  OR "clinical decision support" OR CDSS OR "medication safety")
AND (clinical OR medical OR health))
AND (LIMIT-TO(LANGUAGE,"English"))
AND PUBYEAR > 2013 AND PUBYEAR < 2027
```

7. Notes on adaptation and reproducibility

Field tags and syntax were adapted to each platform so that the retrieved concept sets are equivalent to the base string in Section 2.2. Truncation and controlled-vocabulary (e.g., MeSH) expansion were not used, to keep the strings directly comparable across platforms and reproducible by readers. Phrase terms were retained in quotation marks. The reference lists of key reviews were hand-searched (citation chasing) to capture landmark and very recent (2025–2026) items whose indexing may lag. The overall identification, screening and selection flow is summarised in Figure 1 and Section 2.4 of the main text.
